# Unreferenced spatial localization under monocular and dichoptic viewing conditions

**DOI:** 10.1038/s41598-021-00597-9

**Published:** 2021-11-01

**Authors:** Apoorva Karsolia, Scott B. Stevenson, Vallabh E. Das

**Affiliations:** College of Optometry, University of Houston, 4901 Calhoun Rd, Houston, TX 77204 USA

**Keywords:** Oculomotor system, Saccades, Ocular motility disorders, Vision disorders, Human behaviour

## Abstract

Knowledge of eye position in the brain is critical for localization of objects in space. To investigate the accuracy and precision of eye position feedback in an unreferenced environment, subjects with normal ocular alignment attempted to localize briefly presented targets during monocular and dichoptic viewing. In the task, subjects’ used a computer mouse to position a response disk at the remembered location of the target. Under dichoptic viewing (with red (right eye)–green (left eye) glasses), target and response disks were presented to the same or alternate eyes, leading to four conditions [green target–green response cue (LL), green–red (LR), red–green (RL), and red–red (RR)]. Time interval between target and response disks was varied and localization errors were the difference between the estimated and real positions of the target disk. Overall, the precision of spatial localization (variance across trials) became progressively worse with time. Under dichoptic viewing, localization errors were significantly greater for alternate-eye trials as compared to same-eye trials and were correlated to the average phoria of each subject. Our data suggests that during binocular dissociation, spatial localization may be achieved by combining a reliable versional efference copy signal with a proprioceptive signal that is unreliable perhaps because it is from the wrong eye or is too noisy.

## Introduction

The exploration of the environment around us to detect, discriminate and gather information on pertinent objects is largely driven by visual and oculomotor processes. Since the retinal coordinates of stationary objects shift with every eye movement, an internal representation of space must be developed for coordinated perception and action. In the simple case of a single target in the periphery, the target position relative to the fovea determines the saccade parameters needed to orient to the target^1^. However, the situation gets rapidly complicated when more than one target is presented or if the target is presented transiently. The connection between retinal location and saccade parameters can be dissociated using a “double step paradigm”^2,3^, in which a pair of targets are flashed briefly, and subjects are asked to look at one location and then at the other. Subjects are still able to accurately saccade to the second target, suggesting that the remembered location of the second target has been updated to reflect the change in eye position due to the first saccade.

In the double step paradigm, visual information is not available to update the location of the second target; consequently extra-retinal information is required. Two frequently cited sources of this information for spatial localization are “efference copy” (an internal copy of the saccade command to the first target) and proprioception (sensory input from extra ocular muscle receptors representing eye position)^4^. Efference copy signals have been implicated in maintaining visual continuity across saccades by predictive saccadic remapping of the neuronal receptive field as well as shifting attention gain fields towards the target of interest^5^. Oculomotor proprioception is a non-visual signal representing the stretch response from proprioceptive receptors in the extraocular muscles^6^. Several studies have identified oculomotor proprioception as contributing to spatial localization. Subjects with normal ocular alignment perceive passive changes in eye position in total darkness^7^. Altering proprioceptive signals by mechanical vibration of oculomotor signals produced target illusions in the direction of the deviated eye which synchronized with errors in open loop pointing^8–10^. Lewis et al.^11^ observed that the oculomotor proprioceptive signal is crucial for long term oculomotor calibration of eye movement and eye movement conjugacy, and that this signal is essential for making accurate saccades to multiple remembered locations^12–14^. Studies on rhesus monkeys have further identified the sensory representation of eye position in the primary somatosensory cortex (area 3a)^15^. In a relatively recent study, Poletti and colleagues determined that the accuracy and precision of spatial localization appeared to follow a model based on an optimal combination of retinal (most important contributor when available), efference copy, and proprioceptive signals^16^. In addition, oculomotor proprioception may play a role in stereoscopic slant perception^17^.

Binocular viewing adds a further complication, particularly when a target is presented to one eye and then fixation is acquired by the other eye. It is unclear to what extent proprioception and efference copy may include information about eye alignment, so that spatial locations viewed by one eye can be used to generate a saccade to the remembered target with the other eye. The problem is particularly acute when an individual has a constant eye turn (strabismus), that causes radically different visual directions for a target seen by one eye or the other, but it also applies to normal subjects who have a significant phoria (latent eye deviation). For example, when the binocular view is dissociated, so that diplopia and fusion cues are eliminated, the eyes typically drift apart to a phoria position. The saccade required to fixate a given target location will then vary on which eye is going to fixate it.

The overall goal of the study was therefore to investigate the role of oculomotor efference copy and proprioception on localizing remembered targets in individuals with normal ocular alignment. We assessed the accuracy and precision of localization while subjects performed a spatial localization task under monocular and dichoptic viewing conditions, with flashed targets and no visual references. The task was performed under these viewing conditions to assess how the transfer of oculomotor stretch response and efference copy might affect the internal representation of space. The proposed research is important to understand how this spatial updating mechanism is altered under conditions where the two eyes are dissociated in an environment with no spatial cues.

## Results

**Figure 1:**
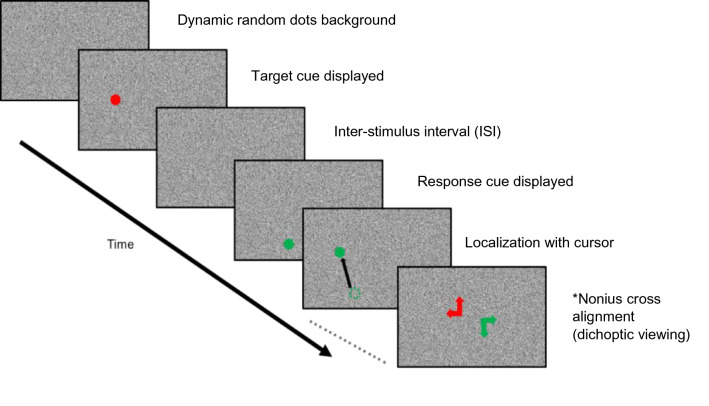
Sequence of events for a trial (monocular or dichoptic paradigm). Two 1.5° diameter circles (red—target cue; and green—response cue) were displayed in sequence, on a random dot background, with a variable inter-stimulus interval (ISI = 0.25, 0.5, 1, 1.5, 2, 5, and 7 s). Subjects then used a mouse cursor (black arrow; not visible during the trial) to mark the remembered location of the target cue. Red and green nonius lines were displayed after every 5th trial during the dichoptic viewing paradigm, and subjects were asked to align the two lines to provide an estimate of the phoria.

The goal of our study was to investigate visual localization during monocular and dichoptic viewing. Ten subjects (23–62 years, four female and six male) with normal ocular alignment and best corrected visual acuity of 20/32 or better in both eyes were included in this study. Nine subjects were tested in the monocular viewing paradigm and six subjects performed the dichoptic viewing paradigm. Paradigm details are in the methods and a time sequence of presentation of target and response cues is shown in Fig. 1. Subject demographics, best corrected visual acuity, and ocular dominance are described in Table 1.

**Table 1:** Demographic data of ten subjects included in the study.

Subject	Gender	Age (years)	Habitual correction	Prescription			Visual Acuity			Dominant eye
				Right eye (D)	Left eye (D)	Add (D)	Right eye	Left eye	Both eyes	
1*	Female	31	None	–	–	–	20/16	20/20	20/20	RE
2*	Male	50	Glasses	− 4.50/− 0.75 × 105	− 4.75/− 0.50 × 95	2.00	20/32	20/20	20/20	LE
3*	Male	62	Trifocal CL	− 2.25	− 3.50	2.50	20/20	20/20	20/20	LE
4	Male	33	Glasses	− 2.25/− 1.0 × 90	− 2.25/− 1.0 × 90	–	20/25	20/25	20/16	LE
5	Female	34	Glasses	− 1.00	− 1.00	–	20/20	20/20	20/20	RE
6	Male	31	Glasses	− 0.50/− 0.50 × 75	− 0.50/− 0.50 × 165	–	20/16	20/16	20/16	RE
7	Female	42	None	Pl/− 1.0 × 30	Plano	1.50	20/25	20/20	20/16	RE
8*	Female	25	None	–	–	–	20/16	20/16	20/16	LE
9*	Male	29	None	− 0.75	− 0.75/− 0.50 × 130	–	20/40	20/32	20/32	LE
10*	Male	22								

Asterisk marks the subjects that performed the dichoptic viewing paradigms. We were unable to collect demographic data on subject 10 due to IRB stipulations associated with minimizing exposure to COVID-19.

*RE* right eye, *LE* left eye.

### Monocular viewing paradigm

This experiment was conducted to evaluate the influence of inter-stimulus interval (ISI) on visual localization of targets presented with no spatial reference. Nine subjects performed the experimental paradigm under monocular right eye viewing. Accuracy of localization was measured as mean horizontal and vertical errors in localization, and the precision of localization was determined by a variance measure (68% bivariate contour ellipse area—BCEA).

**Figure 2:**
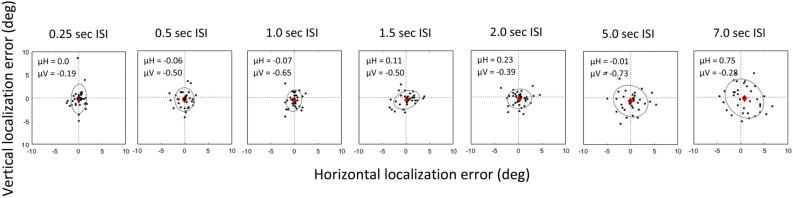
Scatter plot displaying horizontal and vertical localization error across inter-stimulus interval (S6) under monocular viewing conditions. Black dots represent the error in localization for each trial. Red diamond marks the mean horizontal and vertical error in localization across trials providing an estimate of accuracy of localization. BCEA (precision of localization) is the area represented by the grey ellipse.

**Figure 3:**
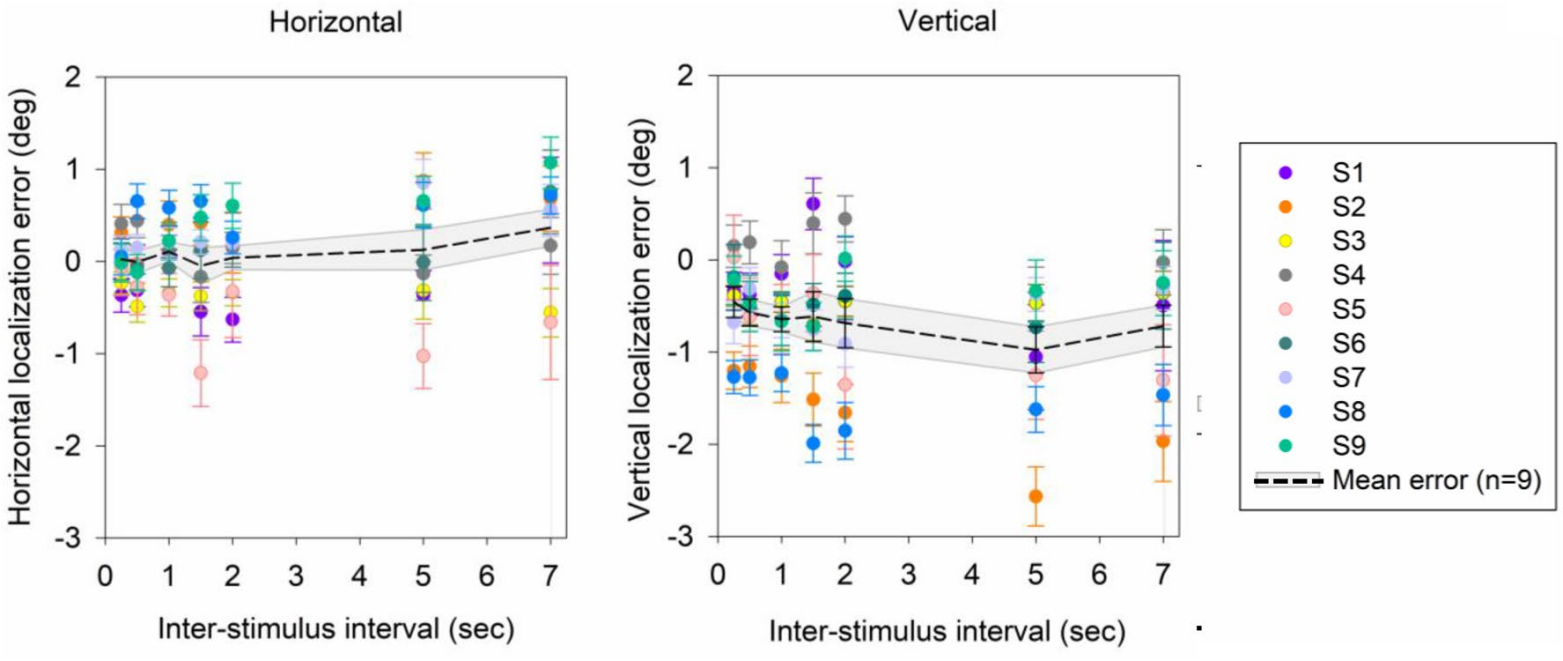
Mean horizontal and vertical localization error across inter-stimulus interval (0.25, 0.5, 1.0, 1.5, 2, 5 and 7 s). Black dashed line represents the mean localization error and grey shading represents the standard error of localization for all subjects (n = 9). Each color represents the mean and standard error of horizontal and vertical error in localization for each subject.

The inter-stimulus interval (ISI) between the target and response cue had a significant effect on precision but not accuracy of horizontal and vertical localization. Figure 2 displays the trial-by-trial localization error across ISIs in one sample subject (S6). The mean horizontal and vertical localization error for short ISIs was generally small and within the dimensions of the target cue (1.5°). Further, the mean horizontal and vertical localization error was not significantly different between 0.5 and 7 s of ISI (Horizontal: 0.5 s: − 0.06° ± 0.23°; 7 s: 0.75° ± 0.45°; p > 0.05 paired t test; Vertical: 0.5 s: − 0.50° ± 0.29; 7 s: − 0.28° ± 0.47; p > 0.05 paired t test). Similarly, when tested as a group, mean horizontal and vertical localization error was not significantly different between 0.5 and 7 s of ISI (Horizontal: 0.5 s: − 0.01° ± 0.12°; 7 s: 0.38° ± 0.20°; p > 0.05 paired t test; Fig. 3; Vertical: (0.5 s: − 0.57° ± 0.15°; 7 s: − 0.72° ± 0.23°; p > 0.05 paired t test; Fig. 3). In summary accuracy of localization did not change significantly with time.

**Figure 4:**
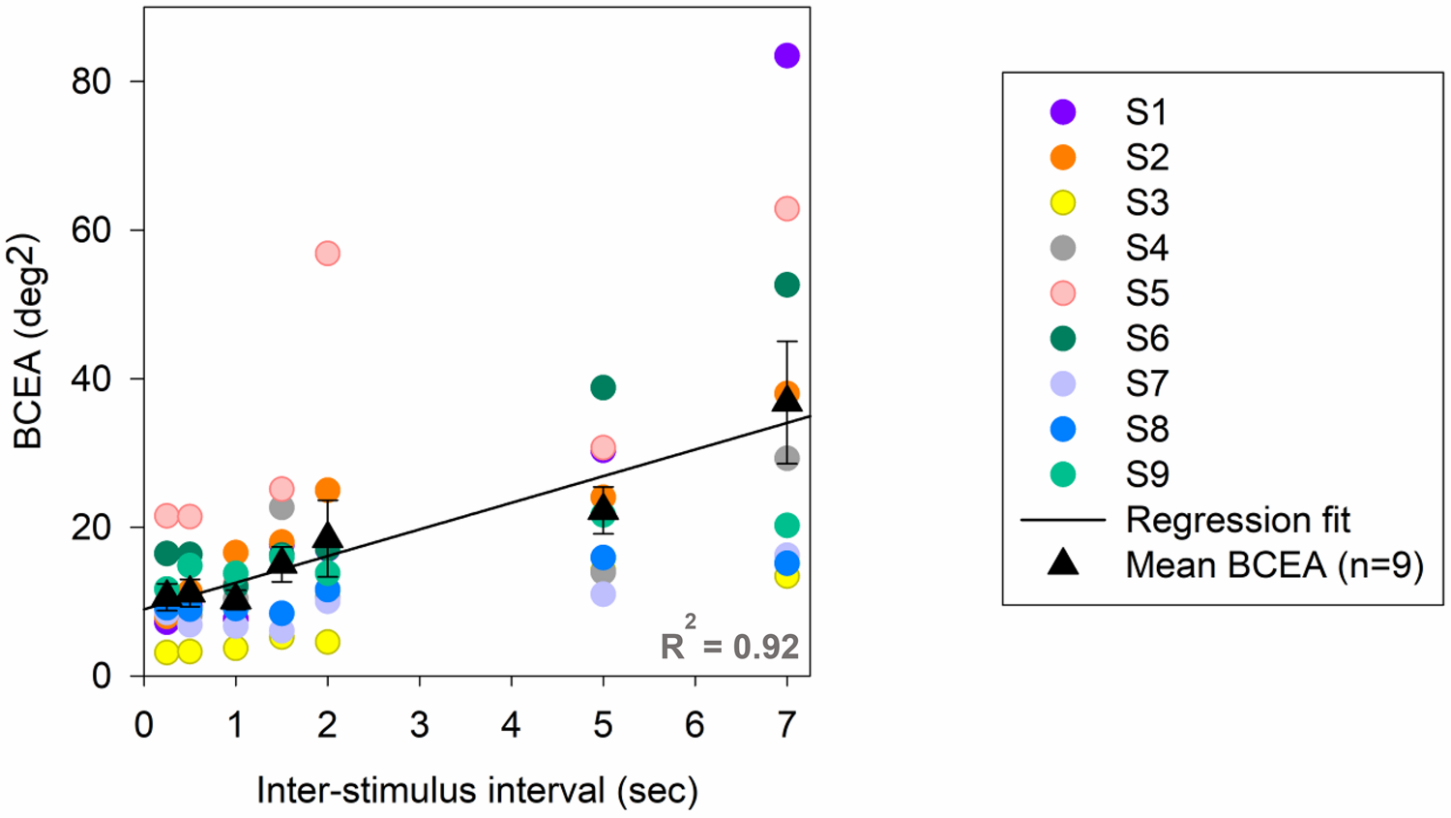
Relationship between BCEA and inter-stimulus interval. Black triangles represent the mean and standard error in BCEA across subjects. (n = 9). Each color represents the BCEA for a single subject. The black line denotes the linear regression fit.

The variability of localization was significantly influenced by inter-stimulus interval. Longer ISIs (> 2 s) resulted in a significantly greater BCEA in all subjects (0.5 s: 11.13 ± 1.64 deg^2^; 7 s: 36.79 ± 7.36 deg^2^; p < 0.05 paired t test; Fig. 4). Within each subject, the rate of change of BCEA with ISI was idiosyncratic; some subjects (S2, S6) showed a rapid and large change in BCEA with increasing ISI while in others (S3, S7), the change was slower or smaller. At shorter ISIs (< 2 s), BCEA was less variable between subjects but as ISI increased, a larger inter-subject variability was observed. We attempted a polynomial linear regression model as well as exponential rise to the maximum model to describe the relationship between BCEA and time. In all subjects, the linear model provided a better, if not similar, fit to an exponential rise to the maximum (R^2^ = 0.92). In summary, the precision of localization worsened with ISI and the rate of change was variable between subjects.

### Dichoptic viewing paradigm

Six subjects, of which five subjects also completed the previous monocular viewing experiment, performed spatial localization under dichoptic viewing conditions. The goal of the dichoptic paradigm was to compare the localization response when the target and response disks were presented to the same-eye (LL, RR) and when the target and response disks were presented to alternate eyes (LR, RL), and to assess the influence of ISI on localization.

**Figure 5:**
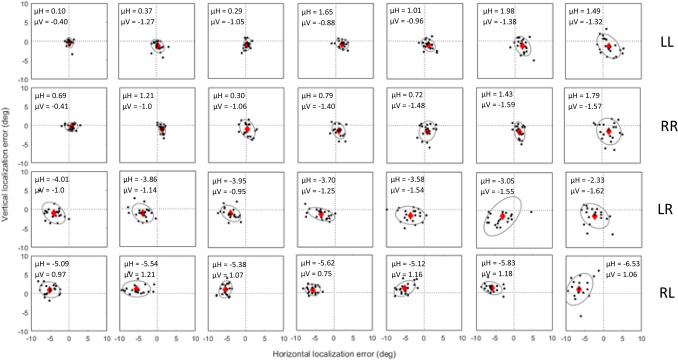
Scatter plot displaying horizontal and vertical localization error across inter-stimulus interval (0.25, 0.5, 1, 1.5, 2, 5, and 7 s left to right) under dichoptic viewing conditions in one subject (S8). LL, RR, LR and RL indicate which eye viewed the target disk and response disk. The bottom two rows show conditions where the disks were seen by alternate eyes (LR and RL). Black dots represent the error in localization for each trial. Red diamond marks the mean horizontal and vertical error in localization across trials. BCEA is represented by the grey ellipse.

Figure 5 displays the results of localization across ISI for LL, RR, LR and RL trials in one sample subject (S8). For all statistical analysis, LL and RR trials were combined as “same-eye” and LR and RL trials were combined as “alternate-eye”. In subject S8, the mean horizontal error was significantly different between 0.5 and 7 s for same-eye trials, (0.5 s: 0.78° ± 0.17°; 7 s: 1.64° ± 0.32°; p < 0.05 paired t test); no significant difference between 0.5 and 7 s ISI was observed for alternate-eye trials (0.5 s: − 4.70° ± 0.37°; 7 s: − 4.39° ± 0.50°; p > 0.05 paired t test), although the magnitude of the errors was much larger than corresponding same-eye trials. For vertical localization error, no significant influence of time was observed for same-eye trials (0.5 s: − 1.14° ± 0.20°; 7 s: − 1.45° ± 0.35°; p > 0.05 paired t test), as well as alternate-eye trials (0.5 s: 0.04° ± 0.30°; 7 s: − 0.27° ± 0.44°; p > 0.05 paired t test).

**Figure 6:**
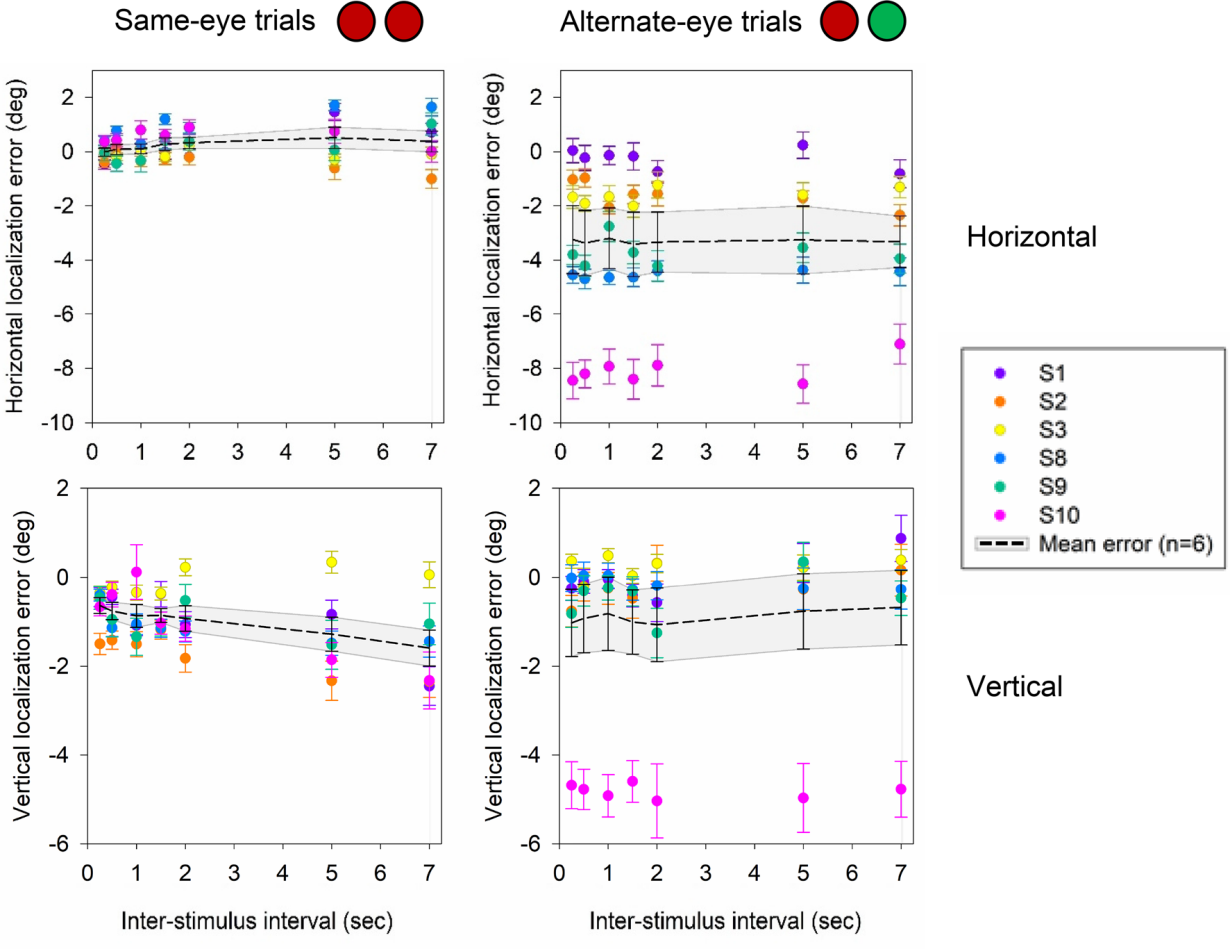
Scatter plot displaying the mean horizontal and vertical localization error across inter-stimulus interval for same eye (LL and RR) and alternate eye (LR and RL) conditions. Black dashed line represents the mean localization error and grey shading represents the standard error of localization for all subjects (n = 6). Each color represents the mean and standard error of horizontal and vertical error in localization for a single subject.

As a group (Fig. 6), the mean horizontal error in localization was not significantly different between 0.50 and 7 s for same-eye conditions (0.5 s: 0.08° ± 0.18°; 7 s: 0.38° ± 0.38°; p > 0.05 paired t test) as well as alternate-eye conditions (0.5 s: − 3.83° ± 1.21°; 7 s: − 3.51° ± 0.97°; p > 0.05 paired t test). Similarly for vertical localization error, no effect of ISI was observed for same-eye trials (0.5 s: − 0.75° ± 0.20°; 7 s: − 1.52° ± 0.39°; p > 0.05 paired t test) as well as alternate-eye conditions (0.5 s: − 0.96° ± 0.80°; 7 s: − 0.68° ± 0.82°; p > 0.05 paired t test).

Dichoptic viewing conditions (same-eye or alternate-eye) had a significant impact on magnitude of localization errors. We observed (Fig. 6) that in general, the mean horizontal error in localization was significantly greater for alternate-eye trials (mean: − 3.30° ± 0.09°) as compared to same-eye trials (mean: 0.23° ± 0.04°, p < 0.05; Signed Rank). Similarly, the mean vertical localization error was significantly greater for alternate-eye trials (mean: − 0.97° ± 0.05°) as compared to same-eye trials (mean: − 0.86° ± 0.07°, p < 0.05; Signed Rank). The interpretation is that binocular dissociation under alternate-eye conditions leads to greater errors in accuracy of localization.

**Figure 7:**
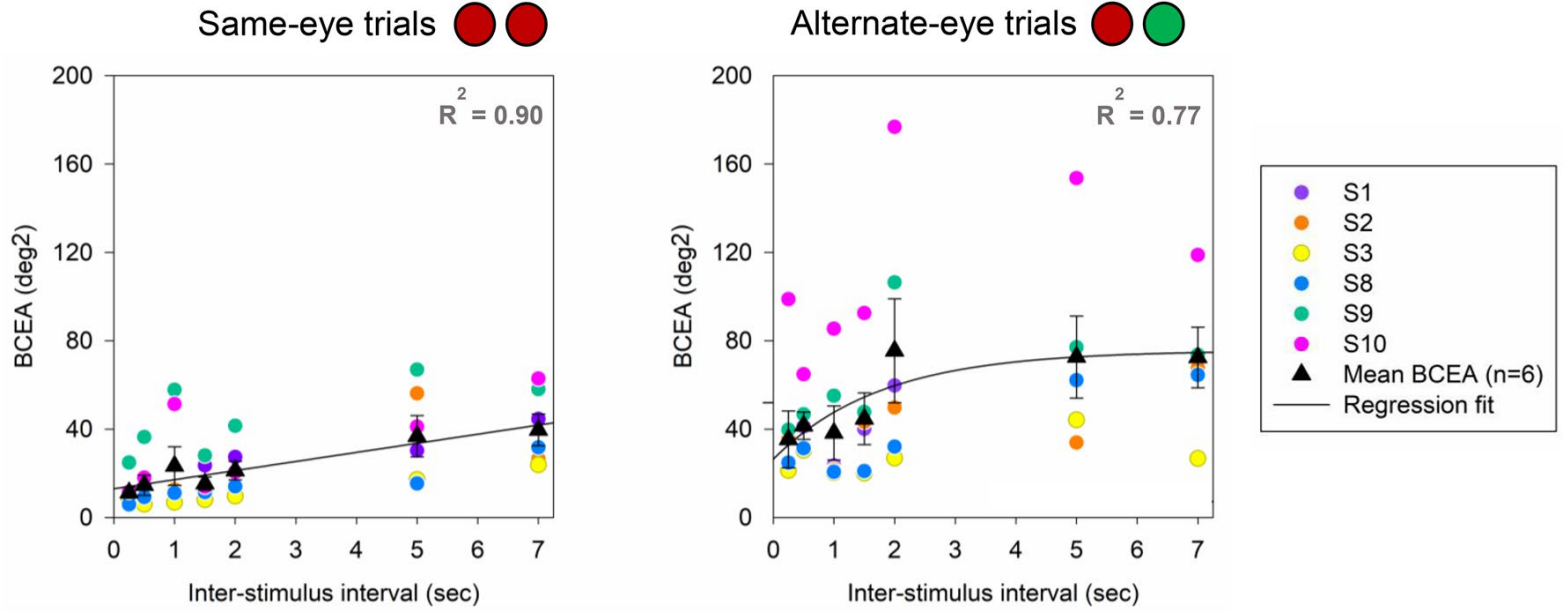
Scatter plot displaying BCEA and relationship with inter-stimulus interval (n = 6) for same-eye and alternate-eye conditions. Black triangles represent the mean and standard error in BCEA across subjects. Each color represents the BCEA for an individual subject. The black line denotes the model fit.

BCEA was computed to assess the influence of dichoptic viewing condition and ISI on variability (precision) of localization error. Overall, BCEA was significantly larger for alternate-eye conditions (mean: 55.45° ± 5.80° deg^2^) as compared to same-eye conditions (mean: 24.09° ± 2.74° deg^2^; p < 0.05 Signed rank test). Comparable to the monocular viewing conditions, we observed a linear increase in BCEA with ISI for same-eye conditions (R^2^ = 0.90; Fig. 7). For alternate-eye conditions, BCEA increased linearly for shorter ISIs (< 2 s) but plateaued for longer ISIs, following the exponential rise to the maximum model (R^2^ = 0.77; Fig. 7).

**Figure 8:**
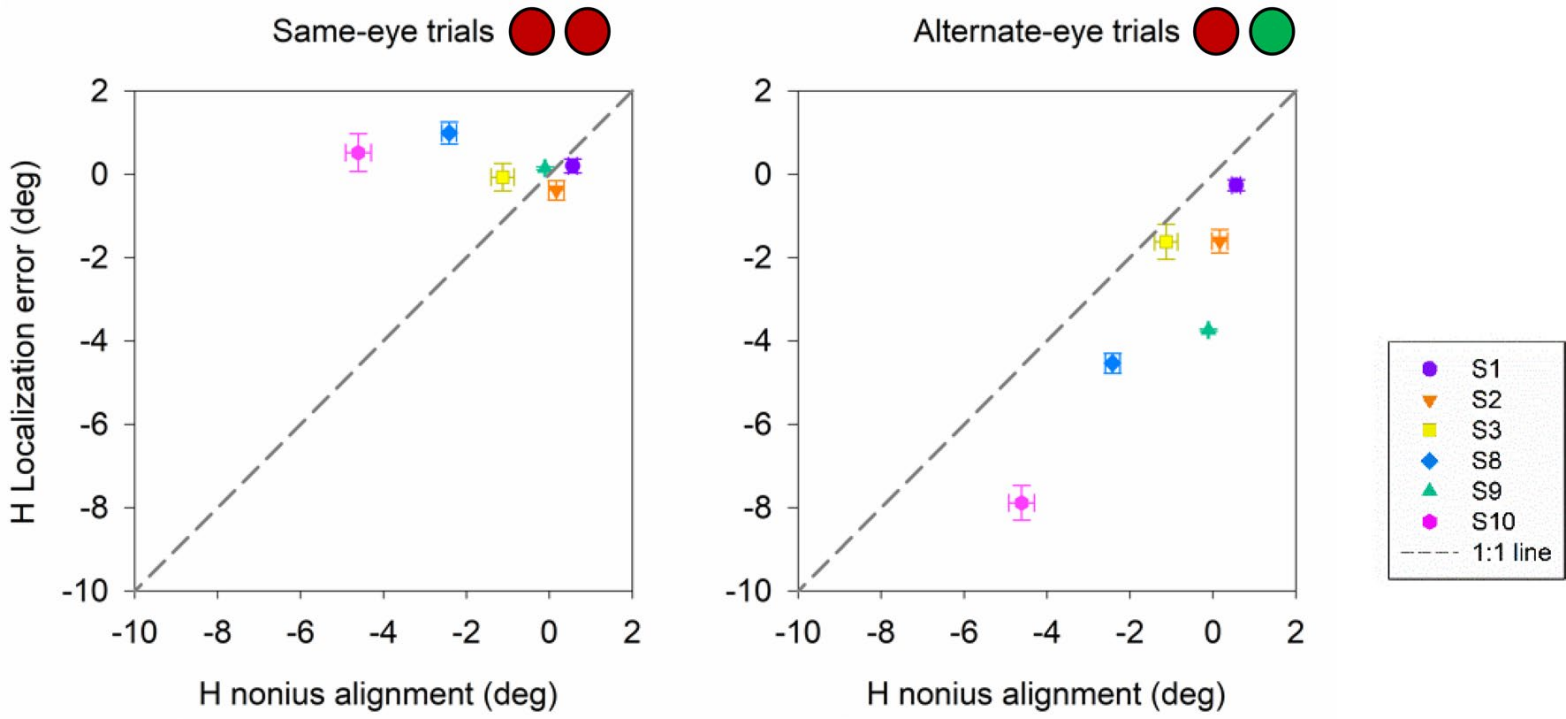
Scatter plot displaying correlation between horizontal nonius alignment and horizontal localization error (n = 6) for same-eye and alternate-eye viewing conditions. Color and shapes represent the mean and standard errors from individual subjects. Grey line is the 1:1 line.

**Figure 9:**
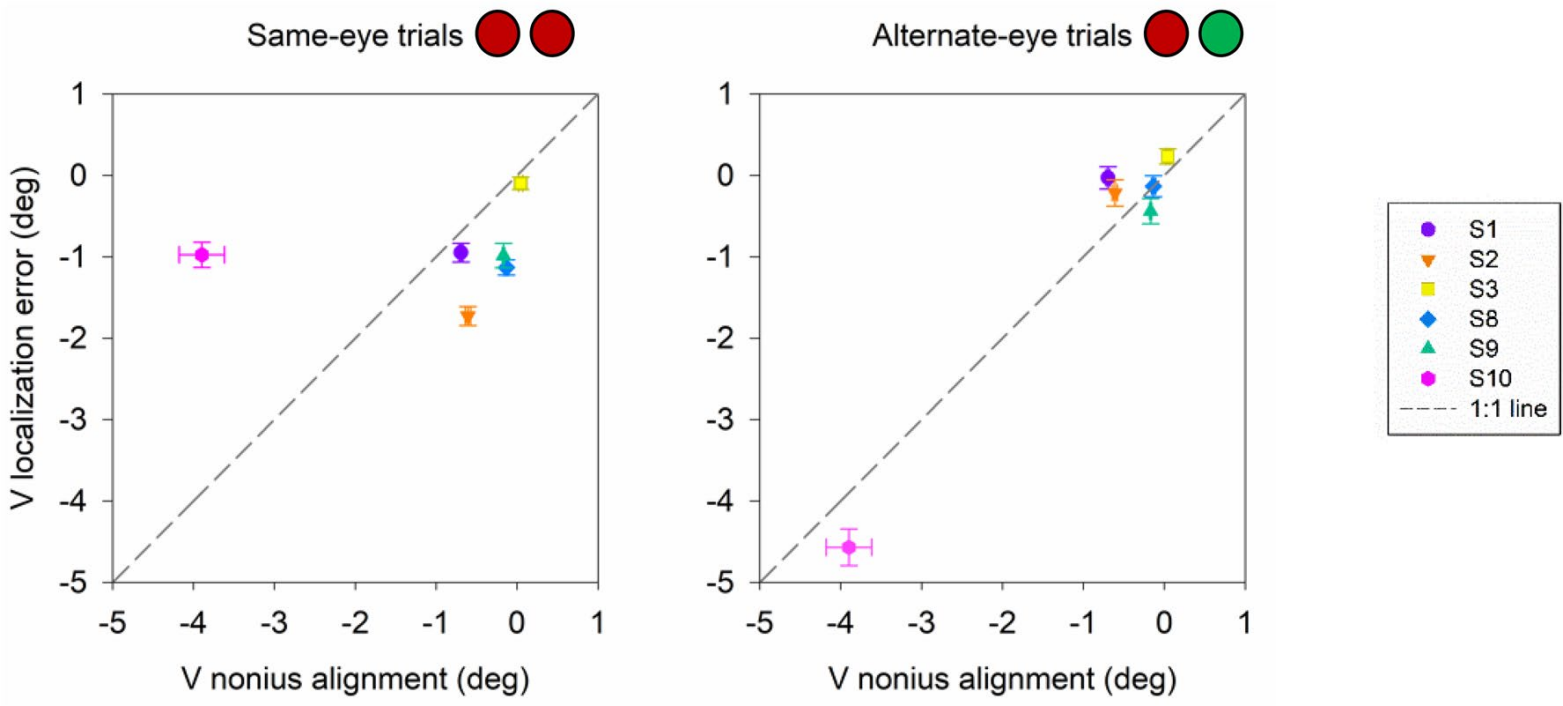
Scatter plot displaying correlation between vertical nonius alignment and vertical localization error (n = 6) for same-eye and alternate-eye viewing conditions. Color and shapes represent the mean and standard errors from individual subjects. Grey line is the 1:1 line.

Binocular dissociation with decorrelated noise and dichoptic targets disrupts fusion mechanisms and phorias typically manifest in subjects with normal ocular alignment. Therefore, we wanted to further evaluate if the larger errors in localization under alternate-eye conditions were dependent on each individual’s phoria. A correlation analysis was performed between average nonius angle and average error in localization in horizontal (Fig. 8) and vertical (Fig. 9) directions. Interestingly, horizontal error in localization correlated strongly with nonius alignment for the alternate-eye condition (p < 0.05, Pearson correlation) but no influence of nonius alignment was observed for the same-eye conditions (p > 0.05, Pearson correlation; Fig. 8). Similarly, vertical error in localization correlated strongly with nonius alignment for the alternate-eye condition (p < 0.05, Pearson correlation) but no influence of nonius alignment was observed for the same-eye conditions (p > 0.05, Pearson correlation; Fig. 9). Note however (Fig. 9), that the relationship between vertical nonius alignment and vertical localization error was influenced significantly by subject S10 (magenta symbol). Removing this outlier data point showed that there was no correlation between vertical nonius alignment and vertical localization error (p > 0.05, Pearson correlation). In summary, Figs. 8 and 9 suggests that the mean error in horizontal localization corresponded strongly to the horizontal phoria recorded during the dichoptic testing procedure.

**Figure 10:**
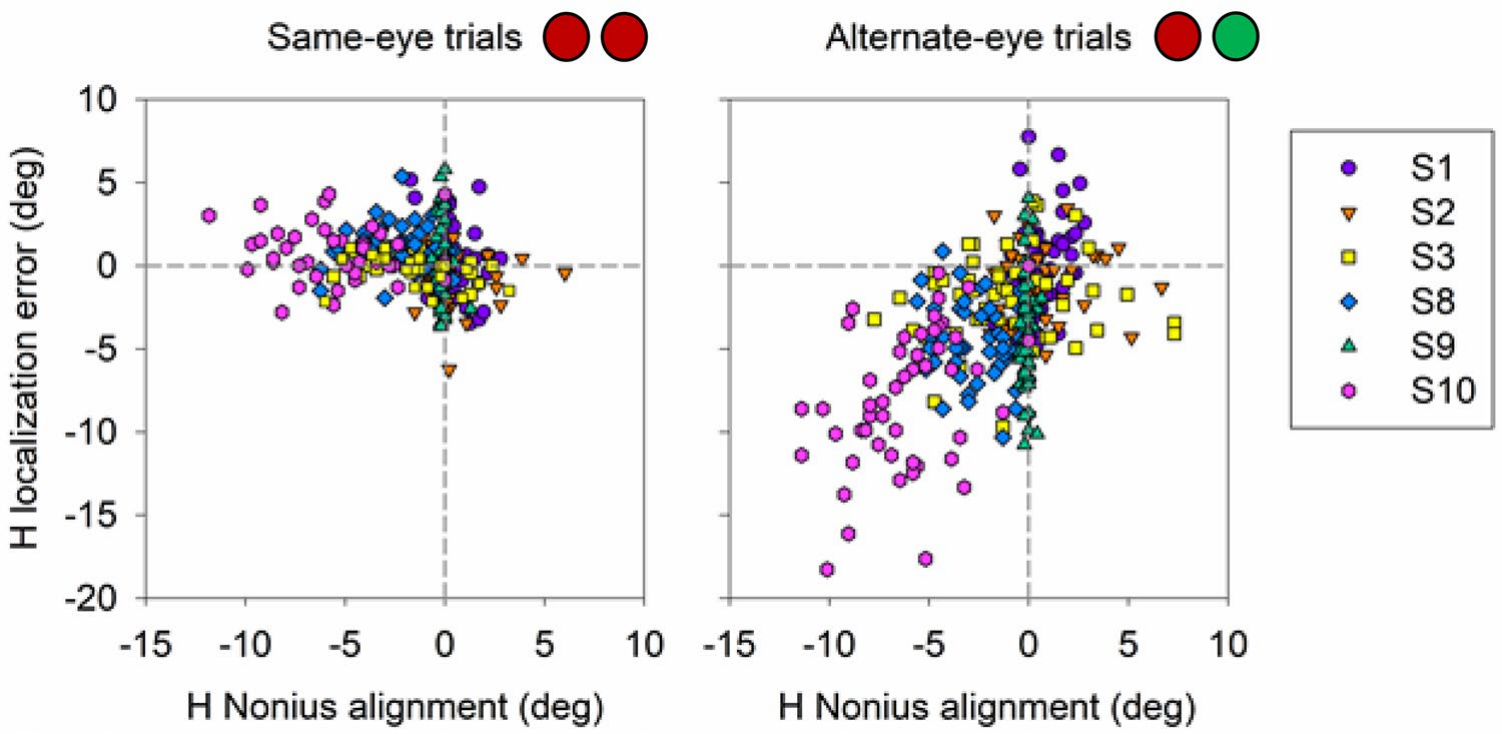
Scatter plot displaying trial by trial correlation, of every 5th trial, between horizontal nonius alignment and horizontal localization error (n = 6) for same-eye and alternate-eye viewing conditions. Each color and shape represents the data for each trial performed by each subject.

**Figure 11:**
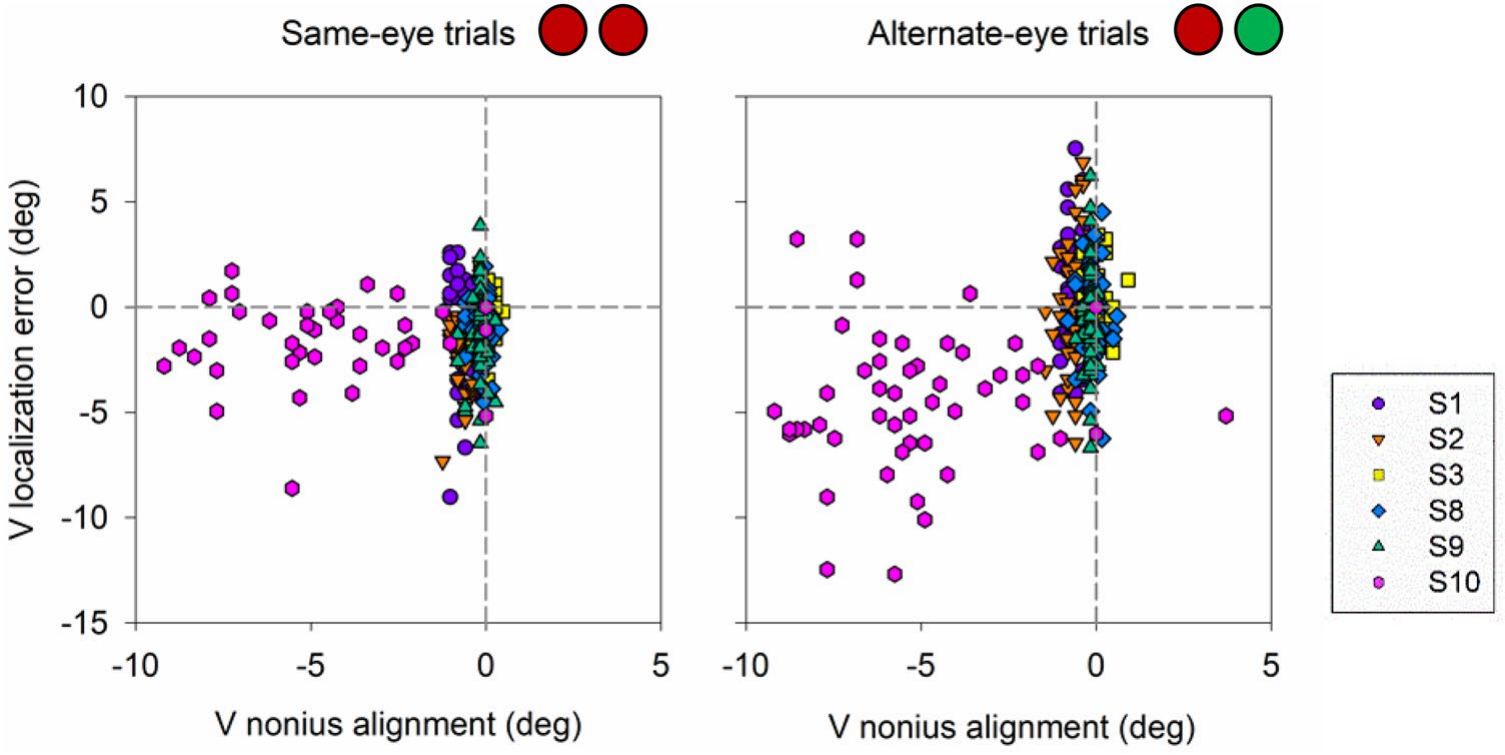
Scatter plot displaying trial by trial correlation, of every 5th trial, between vertical nonius alignment and vertical localization error (n = 6) for same-eye and alternate-eye viewing conditions. Each color and shape represents the data for each trial performed by each subject.

We questioned whether the same relationship between localization error and nonius alignment could be detected on a trial by trial basis, and we found a significant correlation, albeit weak between horizontal error in localization and horizontal nonius error (R = 0.47, p < 0.05, Pearson correlation; Fig. 10) and vertical localization error and vertical nonius error (R = 0.49, p < 0.05, Pearson correlation; Fig. 11) for alternate-eye trials, but no correlation for same-eye trials (p > 0.05, Pearson correlation; Figs. 10, 11). Vertical localization error data during dichoptic viewing was once again skewed due to subject S10; removing the data for this specific subject still resulted in significant but very weak relationship between vertical nonius alignment and vertical localization error (R = 0.12, p < 0.05, Pearson correlation).

## Discussion

The purpose of the study was to investigate factors that influence our ability to localize a remembered target with no spatial reference. The main findings are as follows: (1) in the absence of visual references, the precision of localization but not the accuracy of localization worsens as the inter-stimulus interval between target presentation and target localization increases. (2) Under dichoptic conditions, when the target and response cue were presented to alternate eyes (LR and RL), subjects made greater errors in localization as compared to same-eye condition, (3) errors in localization during the alternate-eye trials correlated with the nonius alignment measured during the trial, suggesting that subjects are unable to compensate for their underlying phoria. (4) Precision of localization during dichoptic viewing decreased as a function of inter-stimulus interval in both same-eye and alternate-eye trials with some differences for longer ISI. Below we discuss each of these findings in greater detail.

Feedback of eye position is critical for online control of eye movements and for higher order tasks such as spatial localization. In the absence of visual reference, eye position information could be provided by efference copy and proprioception signals^18^. Poletti et al.^16^ investigated the relative contributions of efference copy and proprioception to spatial localization by examining the relationship between localization error and the number of intervening saccades between target and response in a paradigm broadly similar to what we used in this study. In their study, they found overall small  mean errors in localization (horizontal: 0.14 ± 0.97; vertical: − 0.03 ± 0.39°) which did not vary with the number of intervening saccades. Although we did not measure eye movements, longer ISI in our study implies larger number of intervening saccades and therefore, our result that mean horizontal and vertical localization errors did not vary with ISI is consistent with their finding.

Using a model-based analysis, Poletti et al. suggested that reliance on efference copy for spatial localization would produce a linear increase in loss of precision with each intervening saccade while using proprioception would result in constant errors^16^. Their group data were best fit with a mixed-model wherein efference copy was used initially (when number of intervening saccades are small) with increasing contribution of proprioception with larger numbers of intervening saccades. In our study, the precision of localization worsened in all subjects as ISI increased, i.e., BCEA increased as a function of ISI. This result is once again in broad agreement with data of Poletti and colleagues with some differences^16^. In our data, we observed some subjects rely on eye position information from efference copy (linear increase with BCEA with time) while other subjects optimized combination of efference copy and proprioception (exponential rise to maximum of BCEA with time). As a group, the multiplicative increase in localization error with ISI (Fig. 4) followed the prediction of the efference copy hypothesis where each eye movement adds its error to the localization response. In our paradigm, while an individual subject could avail of a proprioception signal to aid in spatial localization, the importance of efference copy appears to outweigh that of proprioception. It is certainly possible that the localization response is specific to task or stimulus conditions and differences in the setup between that of Poletti et al. and ours can explain the differences^16^.

We also asked subjects to perform the localization task under conditions of dichoptic viewing where trials were randomized between same-eye and alternate-eye conditions. Errors in localization for same-eye dichoptic conditions mirrored the results of monocular viewing where mean localization error remained constant and BCEA increased with inter-stimulus interval. The interesting finding was that larger mean error in localization was observed during alternate-eye conditions. For monocular and dichoptic same-eye conditions, feedback eye position information from the same-eye is utilized to accurately determine the spatial location of the target. Presumably, efference copy is a single conjugate signal equal to the saccade amplitude and in the case of the same-eye condition, the efference copy signal along with possibly a smaller contribution of proprioceptive feedback from that eye is likely sufficient to keep track of eye position during the ISI and thereafter generate an accurate estimate needed to localize the target in space. On the other hand, in the dichoptic alternate-eye condition, the alternate-eye (eye that did not see the stimulus) has drifted to its phoria location during the ISI and so an efference copy based estimate of location in space would be insufficient because the phoria error is not accounted for.

Consistent with this hypothesis, the greater errors in localization correlated well with phoria recorded during the alternate-eye trials. A decorrelated noise background, such as what we used in our testing, further helps to dissociate the eyes by introducing larger phorias and we found that the shift in localization is in the same direction as the nonius alignment. Previous studies have observed that during monocular viewing^19,20^, if the right eye is occluded and the subject has an exophoria, the stimulus would be displaced to the right and vice versa if the subject has an esophoria. Furthermore, high correlation (0.58–0.77) between individual measures of phoria and target illusion were observed^21^. In our study, correlations between nonius alignment and error were 0.91, suggesting subjects are unable to compensate for their phoria when attempting to localize the target in space.

Efference copy plays an important role in the online updating of eye position and proprioception contributes to the long-term calibration of eye alignment^11^. During dichoptic viewing conditions, eye position information along with a measure of phoria deviation is critical to perform the localization task accurately. Since proprioceptive information arises directly from muscle stretch receptors of each eye, phoria could be transduced accurately via this mechanism. The appearance of localization errors that are correlated to the phoria suggests that the oculomotor system does not effectively use differential stretch response information of eye position from the extraocular muscles of each eye. We therefore postulate that during binocular dissociation, spatial localization is achieved by combining a reliable versional efference copy signal with a proprioceptive signal that is unreliable perhaps because it is from the wrong eye or is too noisy. This agrees with studies on individuals with strabismus^22–24^ who show significantly greater errors in open loop past pointing in the same direction of the deviated eye.

We employed several strategies to ensure that experiments were conducted without any visual spatial reference. Therefore, to identify the effect of non-retinal eye position signals, we ensured that the subject had minimal visual spatial reference by presenting a full field dynamic white noise background comprising of decorrelated random dots. Moreover, the extent and curvature of the screen covered a visual angle of 180° × 90°, and targets were presented within the central 20° of screen. Since spatial reference from peripheral retina has a negligible influence on localization^25^, we expect any peripheral retinal error signal, if available, would be mostly ineffective in providing visual reference for localization. The relatively large precision errors in localization (BCEA), especially for longer ISI, suggest that this is indeed true. We also opted not to measure eye movements since using any kind of eye tracker that was in our possession would have provided spatial cues; instead we relied on time (variable ISI) to investigate errors in localization. Further, subjects performed the task unaided or with contact lens to minimize spatial cues from the spectacle frame. We believe that lack of correction had negligible effect on perception of the cues as the dimensions (~ 1.5°), supra threshold contrast (282% Weber), macular locations within the central ± 10° and close distance (35 cm) from the screen ensured high visibility of the targets and minimized effects due to optical blur. Furthermore, any optical blur would be similar across inter-stimulus interval and would not have a significant effect on the results of the study. The dichoptic viewing paradigm allowed us to investigate how the transfer of eye position information from one eye to the other influences’ localization. For this we ensured that there was no leakage, i.e., red target visible through green lens and vice versa. The luminance through the red lens (2 cd/m^2^) was slightly lower than that through the green lens (3 cd/m^2^), but the subjects were given enough time to adapt to the glasses. Moreover, the results between the monocular and dichoptic same-eye conditions were similar.

## Methods

All experimental procedures were approved by the Institutional Review Board (IRB) at the University of Houston.

### Subjects

Subjects with normal ocular alignment and best corrected visual acuity of 20/25 or better in both eyes were recruited in this study. Preliminary examination involved measurement of visual acuity, ocular alignment (prism bar cover test) and ocular dominance (‘hole in the hand test’). Subjects with ocular and systemic conditions that could impact the individual’s visual and/or localization performance were excluded. Subjects gave verbal and signed informed consent prior to testing, and all procedures were reviewed by the University of Houston IRB and followed the ethical principles of the Declaration of Helsinki.

### Stimuli and testing apparatus

Subjects were seated 35 cm in front of a curved rear projection screen (field of view 180° × 90°) with their head stabilized in a chin/head rest, adjusted to ensure comfort as well as optimal viewing of screen. Test stimuli consisted of two small (~ 1.5° radius) disks (target and response disk) which were presented over a full-field dynamic random dot background. The target and response disk were randomly presented at any of 25 (5 × 5 grid) predetermined locations within the central ± 10°, and the distance between consecutive locations was 5° horizontally and vertically. Since the target locations were within the central ± 10°, we assumed that any difference between the curved screen and horopter was negligible. The stimuli and background were rear projected on the screen using a gamma corrected NEC VE281X projector with a 1024 × 768 pix viewing area and ~ 4.65 pix/° calibration at 35 cm. A correction was made to ensure uniform luminance of 20.32 cd/m^2^ across the curved screen. In certain experiments involving dichoptic viewing, the subjects viewed through red/green glasses that reduced the luminance, on average, by a factor of 0.1. Stimuli and background were generated using MATLAB Psychophysics toolbox (MathWorks, MA). Since the overall goal of the study was to study visual localization in the absence of retinal error information, the experimental setup (large field of view and full-field random dot background) was deliberately developed to eliminate potential spatial reference cues. Further, to avoid visual reference associated with spectacle wear, subjects were uncorrected or were wearing contact lenses while performing the experimental paradigm. All subjects reported being able to clearly see the target and response disks necessary to perform the task.

### Experimental procedure

#### Monocular paradigm

These experiments were performed under monocular right eye viewing with left eye patched. Figure 1 displays the sequence of events within each trial. Specifically, each trial started with the presentation of the dynamic noise background. After 0.5 s, a target disk was flashed for 0.5 s at a random location within the central ± 10°. After an inter-stimulus interval (ISI) of 0.25, 0.5, 1, 1.5, 2, 5, or 7 s, a response disk was presented at a different random location within the central ± 10°. During the delay period subjects were free to make eye movements although they were instructed to attempt to remain within the central ± 10° of the screen. When the response disk was presented, subjects were instructed to fixate on the disk and thereafter use a mouse to move the response disk to the remembered location of the target disk. Specifically, the subject memorized the location of the flashed target cue and used a mouse to localize it without any target reference. Subjects indicated their setting by clicking the mouse button, which also then initiated the next trial. To minimize cues for localization, the mouse cursor was not visible during each trial. The location of the target disk, inter-stimulus interval and the location of the response disk were all randomized for each trial. A total of 210 trials (30 trials for each ISI all shuffled) were collected for each subject.

#### Dichoptic paradigm

The goal of the second experiment was to investigate spatial localization when the target and response disks are presented to the same-eye or to different (alternate) eyes (left eye sees target and right eye sees response disk or vice-versa). To manipulate presentation of the target and response disks to a specific eye, trials were performed under dichoptic viewing with red-green glasses (Right eye—red; Left eye—green), and the target and response disks could be presented to either right eye or left eye leading to four conditions [green target–green response cue (LL), green–red (LR), red–green (RL), and red–red (RR)]. Thus, LL and RR were conditions where the target and response disks are presented to the same-eye (similar to the monocular paradigm) and RL and LR are conditions where target and response disks are presented to alternate eyes. The background random dots were independently generated for red and green colors so that the eyes saw binocularly uncorrelated noise with no identifiable depth plane or stationary features, and therefore did not have a particular disparity. For these experiments, the RGB values of bright and dark areas of each eye’s image were adjusted to ensure that the right eye image was not visible through the left eye and vice versa. Other aspects of the trial including target and response disk locations and inter-stimulus intervals were same as those used for the monocular viewing experiment. Data were collected over 15–20 blocks with each block containing a randomized set of ISIs (0.25, 0.5, 1, 1.5, 2, 5, or 7 s) for each of the target-response cue conditions (LL, LR, RL and RR) i.e., 28 trials per block; for a total of 420–560 trials per subject. Because the dynamic background had no fixed depth plane and no other objects were visible, subjects tended to drift to their phoria posture. To dynamically assess ocular alignment under decorrelated binocular noise, subjects were instructed to align one half (red) of a nonius cross to the green reference half, which was presented at a randomized location within the central ± 10° grid after every 5th trial (Fig. 1).

### Data analysis

Horizontal and vertical errors in localization were calculated as the difference between the reported and actual positions of the target cue for each trial. For each subject, trials with same ISIs and viewing conditions (monocular, dichoptic LL, LR, RL, or RR) were pooled. Precision of localization was quantified as the area of the 68th percentile bivariate contour ellipse (BCEA)^26^. $BCEA = 2.291\pi *\sigma {\text{x}}*\sigma {\text{y}}*\sqrt {1 - p^{2} }$ where, σ_x_ is the SD of horizontal localization error and σ_y_ is the SD of vertical localization error and *p* is the Pearson product moment correlation coefficient of horizontal and vertical localization. To determine the relationship of BCEA with time, the data were fit with linear regression and exponential rise to maximum models.

For trials performed under dichoptic viewing, nonius alignment was calculated on every 5th trial as the difference between nonius half viewed by left eye (green) and right eye (red). The influence of ISI, nonius alignment, and viewing condition on localization error was assessed (SigmaPlot V12.5, Systat Software Inc., San Jose) by performing parametric and non-parametric tests.
